# Crizotinib and PARP inhibitors act synergistically by triggering apoptosis in high-grade serous ovarian cancer

**DOI:** 10.18632/oncotarget.27363

**Published:** 2019-12-10

**Authors:** Irem Durmaz Sahin, Jenny-Maria Jönsson, Ingrid Hedenfalk

**Affiliations:** ^1^Division of Oncology and Pathology, Department of Clinical Sciences, Lund University and Skåne University Hospital, Lund, Sweden; ^2^School of Medicine, Koç University, Istanbul, Turkey

**Keywords:** HGSOC, c-Met, PARP inhibitor, apoptosis

## Abstract

High-grade serous ovarian cancer (HGSOC) is the predominant and most lethal histological type of epithelial ovarian cancer. During the last few years, several new treatment options with PARP inhibitors have emerged. The FDA has approved the PARP inhibitor olaparib (Lynparza™) as maintenance treatment after first-line platinum-containing chemotherapy and olaparib, niraparib (Zejula™) and rucaparib (Rubraca™) are approved as maintenance therapies in the recurrent, platinum-sensitive setting; nevertheless, development of resistance limits their efficacy. In this study, new combinatorial treatment strategies targeting key signaling pathways were explored to enhance the activity of PARP inhibitors in HGSOC. Carboplatin, olaparib, niraparib, the PI3K inhibitor LY294002 and the c-Met inhibitor crizotinib were used for this investigation. PARP inhibitors and carboplatin alone and in combination caused accumulation of DNA double-strand breaks and G2/M cell cycle arrest. In contrast, crizotinib alone or in combination with PARP inhibitors induced accumulation of cells in sub-G1. Crizotinib together with either of the PARP inhibitors was more strongly synergistic than combinations with a PARP inhibitor and carboplatin or the PI3K inhibitor. Sequential combination of crizotinib and a PARP inhibitor resulted in activation of ATM/CHK2 and inhibition of c-Met pathways, contributing to a decrease in RAD51 levels and induction of caspase-3 dependent apoptotic cell death and suggesting that the combination of crizotinib with a PARP inhibitor may be considered and further explored as a new therapeutic strategy in HGSOC.

## INTRODUCTION

Epithelial ovarian cancer (EOC) is the seventh most common malignancy diagnosed in women. With a relative five-year survival less than 50%, it is the fifth most common cause of cancer-related deaths [1, 2]. Cytoreductive surgery and post-operative platinum-based chemotherapy are the standard treatments for patients diagnosed with advanced-stage disease [3–5]. Due to the poor prognosis, associated with a high recurrence rate of >75%, it is vital to investigate new treatment strategies [3]. EOC constitutes five main histological types, of which high-grade serous ovarian cancer (HGSOC) is the most common [6, 7]. In general, HGSOC initially responds well to chemotherapy, but in the majority of cases, chemo-resistance develops due to high proliferative rates and accumulation of genomic aberrations. Mutations in the tumor suppressor gene *TP53* occur in all HGSOCs, in addition to a high degree of chromosomal instability and amplification of genes such as *PIK3CA* [7–9]. Homologous recombination DNA repair pathway deficiency is observed in almost 50% of HGSOCs, approximately 30% of which is due to *BRCA1* or *BRCA2* deficiency [7, 8]. Loss of *BRCA1/2* function in HGSOC is mainly due to germline/somatic mutations or epigenetic modifications [7, 8, 10].

Poly(ADP-ribose) polymerase (PARP) is a fundamental element of the DNA repair pathway, which functions by recognizing single-strand DNA (ssDNA) breaks and activates the base excision repair (BER) pathway [11–14] to resolve these defects in the DNA. Alternatively, when a double-strand (dsDNA) break occurs in the DNA, it is repaired either by error-free homologous recombination (HR) or error-prone non-homologous end joining (NHEJ) [14–17]. BER is responsible for rescuing dsDNA breaks in cells with HR deficiency due to BRCA1/2 loss. When PARP is inhibited in an HR deficient (*BRCA1/2* mutated) cell, neither BER nor NHEJ can repair the ssDNA breaks [17, 18]. Induction of PARP trapping and subsequent replication fork collapse are other action mechanisms of PARP inhibitors [19]. All these mechanisms lead to the development of synthetic lethality in *BRCA1/2* deficient cancers following PARP inhibitor treatment, and several PARP inhibitors including olaparib (Lynparza™), niraparib (Zejula™) and rucaparib (Rubraca™) have now been approved by the FDA and/or the European Medicines Agency for the maintenance treatment of platinum-sensitive, recurrent HGSOC with or without *BRCA1/2* mutations [14, 20–24]. However, similar to many other targeted agents, the efficacy of PARP inhibitors is limited by the development of resistance [25–27]. In this study, new combinatorial treatment strategies aimed at prolonging the anti-cancer activity of PARP inhibitors in HGSOC were investigated.

The PI3K/AKT/mTOR signaling pathway is important for many cellular processes such as proliferation, survival and angiogenesis and multiple genetic aberrations in genes involved in this pathway have been characterized in EOC [3, 28, 29]. These observations encourage exploring the use of PI3K/AKT/mTOR inhibitors for the treatment of EOC. It was also proposed that activation of the PI3K/AKT/mTOR pathway may be responsible for the development of chemotherapy resistance [30]. Furthermore, negative regulation of AKT by BRCA1 together with the proposal that *BRCA1* deficient tumors have aberrant PI3K/AKT signaling suggests that the combination of PARP and PI3K/AKT/mTOR inhibitors may be effective to overcome tumorigenesis and resistance. Previous studies have shown that inhibition of the PI3K pathway in *BRCA1* deficient breast cancer cells increases their sensitivity to PARP inhibitors [31–34]. Therefore, in this study we investigated the combinatorial effect of the PI3K inhibitor LY294002 with PARP inhibitors.

The mesenchymal-epithelial transition factor (c-*MET*) is a proto-oncogene that encodes a receptor tyrosine kinase, with important functions in cell proliferation, invasion, motility and survival [35]. Overexpression or mutation of c-*MET* is observed in many cancer types including liver, ovarian and pancreatic cancer. c-*MET* expression is observed in 70% of ovarian carcinomas, 30% of which present with overexpression. Moreover, it was suggested that c-Met may contribute to the aggressive behavior of ovarian cancer and it has been shown to harbor prognostic information [35–40]. There are several studies proposing that c-Met inhibitors may enhance the activity of PARP inhibitors, and may also be effective in overcoming treatment resistance in other tumor types [41, 42]. Therefore, we investigated the possible synergistic effects of the c-Met inhibitor crizotinib and PARP inhibitors in HGSOC.

We hypothesized that sequential combination of crizotinib or LY294002 with a PARP inhibitor may increase the potency of PARP inhibition. The effect of combining carboplatin and PARP inhibitors was also investigated to compare with the effects of the c-Met and PI3K targeted drugs. Our results indicate that combining a c-Met and a PARP inhibitor significantly enhances the effect of PARP inhibition, thus presenting a new therapeutic strategy in HGSOC.

## RESULTS

### Evaluation of the cytotoxic effects of the drugs on HGSOC cells

**Figure 1:**
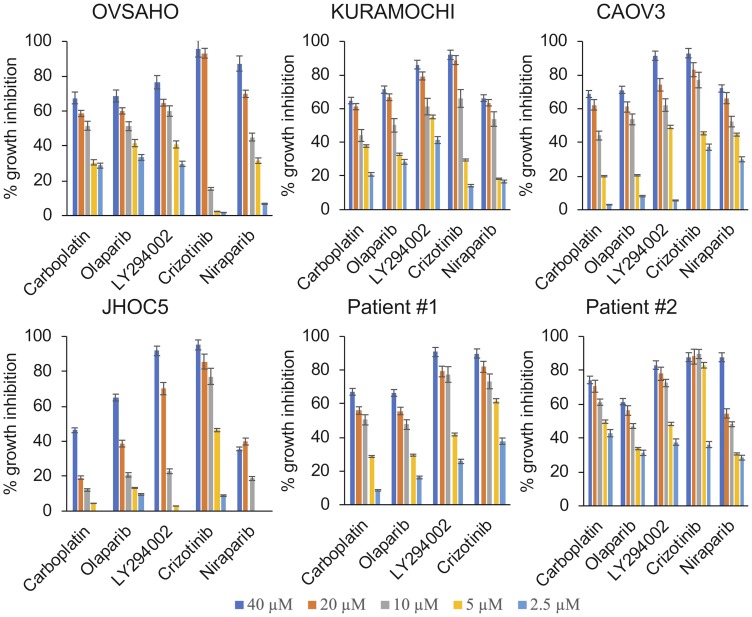
Cytotoxic and growth inhibitory effects of single agent treatments. The HGSOC cell lines KURAMOCHI, OVSAHO and CAOV3, the OCCC cell line JHOC5 and primary cells obtained from ascites from patients diagnosed with HGSOC were treated with increasing concentrations of the respective drugs (2.5 µM–40 µM) for 1 week and the growth inhibitory effects were analyzed using the NCI-SRB Assay.

The cancer cell lines and primary cells obtained from the ascites of two patients diagnosed with *BRCA1/2* wild type HGSOC were treated for 1 week. The NCI-SRB assay revealed that HGSOC cells were sensitive to all treatments while the ovarian clear cell cancer (OCCC) cells (control) were highly resistant to carboplatin and PARP inhibitors. Cells from patient #1 showed a response pattern similar to the HGSOC cell lines (Figure 1, Table 1). Primary cells from patient #2 however had a very low IC_50_ for carboplatin, mirroring the fact that platinum therapy was stopped for the patient due to toxicity (Figure 1, Table 1). Growth inhibitory effects of the drugs were also analyzed in real-time using the xCelligence real-time cell electronic sensing system (Table 2). No significant difference in PARP inhibitor sensitivity was observed between the *BRCA1/2* wildtype CAOV3 cell line and the *BRCA2* deficient KURAMOCHI and OVSAHO cell lines (Figure 1, Table 1).

**Table 1:** IC_50_ (µM) concentrations with single agent treatments (NCI-SRB assay)

	OVSAHO	KURAMOCHI	CAOV3	JHOC5	Patient #1	Patient #2	MCF10A
Carboplatin	12 ± 2.5	13.4 ± 1.3	15.1 ± 3.7	44.4 ± 6.6	14.8 ± 3.3	4.8 ± 0.4	199.5 ± 20.3
Olaparib	9.3 ± 0.2	10.1 ± 1.4	13.5 ± 0.4	24.2 ± 4.7	14.7 ± 4.2	13.9 ± 3.1	30.5 ± 12.2
LY294002	8 ± 3.2	5.1 ± 0.8	8.4 ± 1.3	15.5 ± 2.9	5.9 ± 1.1	4.5 ± 1.5	12.4 ± 2.8
Crizotinib	6.9 ± 1.1	4 ± 2.5	4.5 ± 1.3	6.7 ± 0.5	4.2 ± 0.2	1.8 ± 0.6	4.56 ± 0.9
Niraparib	10.5 ± 0.4	13.3 ± 3.0	9 ± 2.4	97 ± 10.0	N/A	10 ± 2.2	78.2 ± 11.4

N/A, not available.

**Table 2:** Real-time IC_50_ (µM) concentrations with single agent treatments (xCeLLigence real-time cell sensoring)

	OVSAHO		KURAMOCHI		CAOV3	
	72 hr	168 hr	72 hr	168 hr	72 hr	168 hr
Carboplatin	42.1 ± 8.4	12.4 ± 4.5	37.2 ± 7.4	13.7 ± 3.3	36.3 ± 9.4	16.7 ± 2.4
Olaparib	33.6 ± 10.1	9.9 ± 3.3	36.8 ± 3.4	9.8 ± 1.2	23.1 ± 7.6	17.6 ± 1.1
LY294002	6.9 ± 3.6	5 ± 0.2	8.7 ± 1.1	7.4 ± 0.4	7 ± 1.4	6.1 ± 0.3
Crizotinib	6 ± 1.8	6.5 ± 2.0	6.8 ± 0.4	7.6 ± 0.6	6.9 ± 1.1	6.1 ± 0.9
Niraparib	29.9 ± 2.2	13.3 ± 1.4	25.7 ± 6.2	14.6 ± 1.8	23.1 ± 0.3	9.6 ± 1.2

### c-Met inhibition is synergistic with PARP inhibition

**Figure 2:**
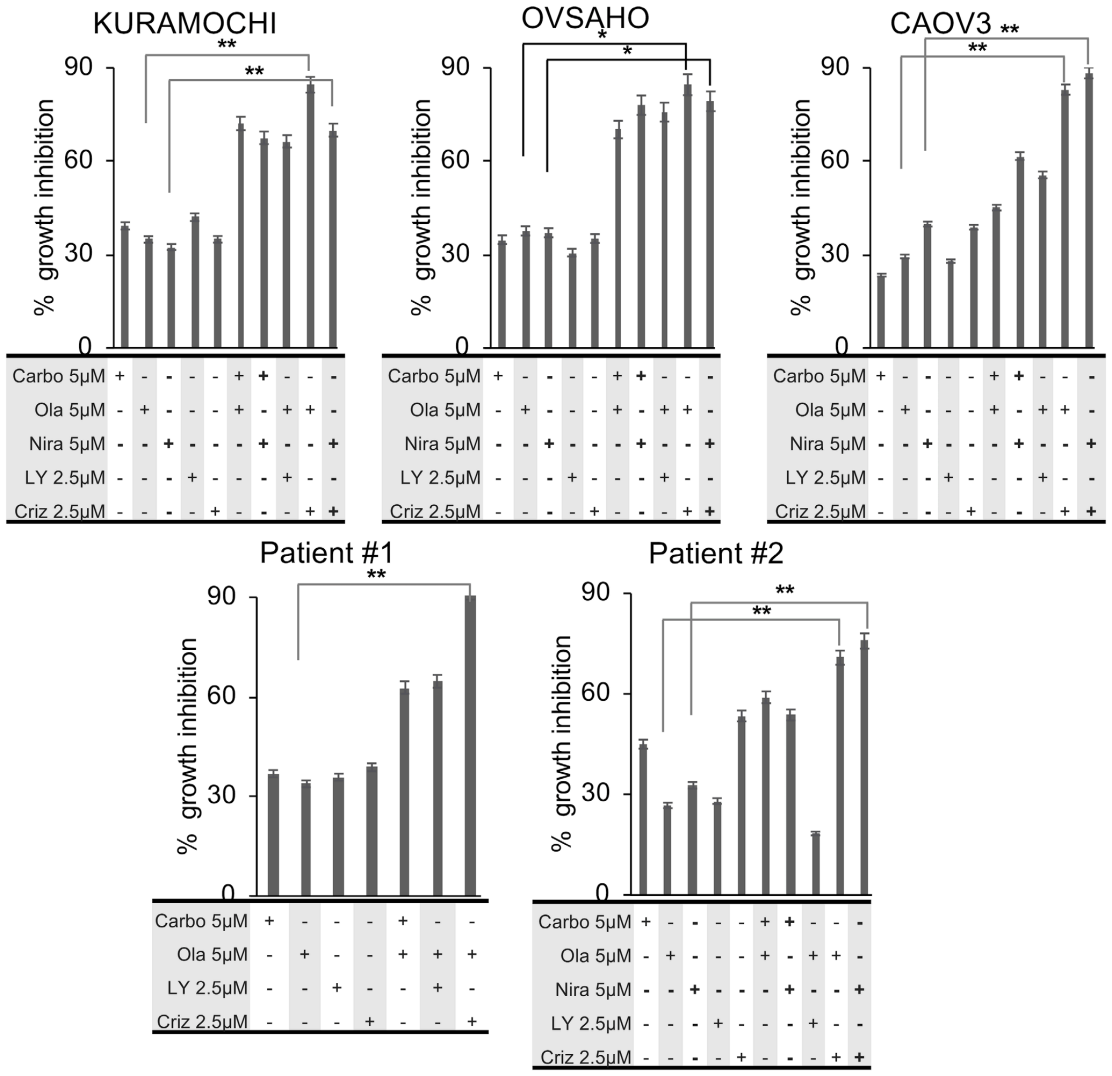
Growth inhibitory effects of sequential combination treatments. HGSOC cells were treated sequentially for 1 week and growth inhibitory effects were measured by the NCI-SRB assay. All experiments were conducted in triplicates. Statistical analysis of mean values (*n* = 3) was performed with student’s *t*-test (^*^ *p* < 0.05; ^**^ *p* < 0.01; ^***^ *p* < 0.005).

In order to assess the potential synergistic effects of the drugs we investigated the growth inhibitory effects at sub-optimal doses of the respective drugs (doses < IC_50_ values). HGSOC cells were treated with the drugs at sub-optimal doses, either as monotherapies or in sequential regimens. The conditions with the highest synergistic potential were selected for further analyses. In all three HGSOC cell lines, treatment with carboplatin, olaparib, niraparib and LY294002 resulted in 20–30% growth inhibition at 5 µM concentrations, while 20–30% growth inhibition was observed at 2.5 µM for crizotinib. When administered sequentially at IC_20–30_, carboplatin and either of the PARP inhibitors increased the growth inhibition to 50–70%. The combination of LY294002 and olaparib at IC_20–30_, also induced a 50–70% growth inhibition. When crizotinib was combined with the PARP inhibitors at IC_20–30_ doses, growth inhibition was increased to 80–90% (Figure 2). Moreover, increased sensitivity of the CAOV3 cells to the combination of carboplatin and either of the PARP inhibitors was observed, which may be due to these cells harboring an *ATM* mutation [43]. The CI values suggest that sequential combinations of crizotinib with olaparib or niraparib were more synergistic compared to the combination of either carboplatin or LY294002 with the PARP inhibitors (Table 3). Importantly, *in vitro* results from primary human ascites-derived cancer cells were consistent with the cell line experiments (Figure 2, Table 3).

**Table 3:** Calculation of combination indices (CI values) of the sequential combination treatments for 1 week

CI values					
	KURAMOCHI	OVSAHO	CAOV3	Patient #1	Patient #2
Carboplatin 5 µM + Olaparib 2.5 µM	0.93	0.80	0.96	1.00	2.85
Carboplatin 5 µM + Olaparib 5 µM	**0.84**	**0.84**	**1.02**	0.90	1.01
Carboplatin 2.5 µM + Olaparib 2.5 µM	0.96	0.94	0.96	0.93	1.50
Carboplatin 2.5 µM + Olaparib 5 µM	0.91	0.90	1.22	1.36	5.48
Carboplatin 5 µM + Niraparib 2.5 µM	1.09	1.01	0.99	N/A	1.10
Carboplatin 5 µM + Niraparib 5 µM	**0.88**	**0.80**	**0.89**	N/A	1.17
Carboplatin 2.5 µM + Niraparib 2.5 µM	2.16	1.32	1.14	N/A	0.47
Carboplatin 2.5 µM + Niraparib 5 µM	1.91	1.12	1.55	N/A	1.23
Olaparib 5 µM + LY 2.5 µM	**0.94**	**0.91**	**0.88**	0.89	2.58
Olaparib 5 µM + LY 5 µM	1.00	0.97	0.95	0.26	1.23
Olaparib 2.5 µM + LY 2.5 µM	1.00	0.98	0.93	1.31	1.35
Olaparib 2.5 µM + LY 5 µM	1.05	1.01	1.13	1.26	1.14
Crizotinib 5 µM + Olaparib 5 µM	0.74	0.75	0.69	0.79	1.06
Crizotinib 5 µM + Olaparib 2.5 µM	0.73	0.69	0.63	0.77	1.04
Crizotinib 2.5 µM + Olaparib 5 µM	**0.68**	**0.70**	**0.68**	0.65	0.93
Crizotinib 2.5 µM + Olaparib 2.5 µM	0.86	0.70	0.62	0.61	1.15
Crizotinib 5 µM + Niraparib 2.5 µM	0.80	0.75	0.74	N/A	1.21
Crizotinib 5 µM + Niraparib 5 µM	0.81	0.81	0.77	N/A	1.07
Crizotinib 2.5 µM + Niraparib 2.5 µM	0.98	0.76	0.71	N/A	0.91
Crizotinib 2.5 µM + Niraparib 5 µM	**0.80**	**0.74**	**0.71**	N/A	0.91

CI values in bold indicate the drug concentrations selected for further experiments, sd < 3%.

N/A, not available.

In order to test the toxicity in normal cells, we used the non-tumorigenic breast epithelial cell line MCF10A. The results indicated that carboplatin had no effect on viability. On the other hand, the IC_50_ values for PARP inhibitors were much higher than in the cancer cell lines or patient samples, indicating that these drugs are less toxic to normal cells than to HR deficient cancer cells. The effect of crizotinib in MCF10A was however similar to cancer cells, suggesting that crizotinib may display toxicity towards normal cells (Supplementary Figure 1, Table 1). Importantly however, when we introduced the combination regimen to MCF10A cells, no synergistic effect was observed.

### Accumulation of DNA double strand breaks

**Figure 3:**
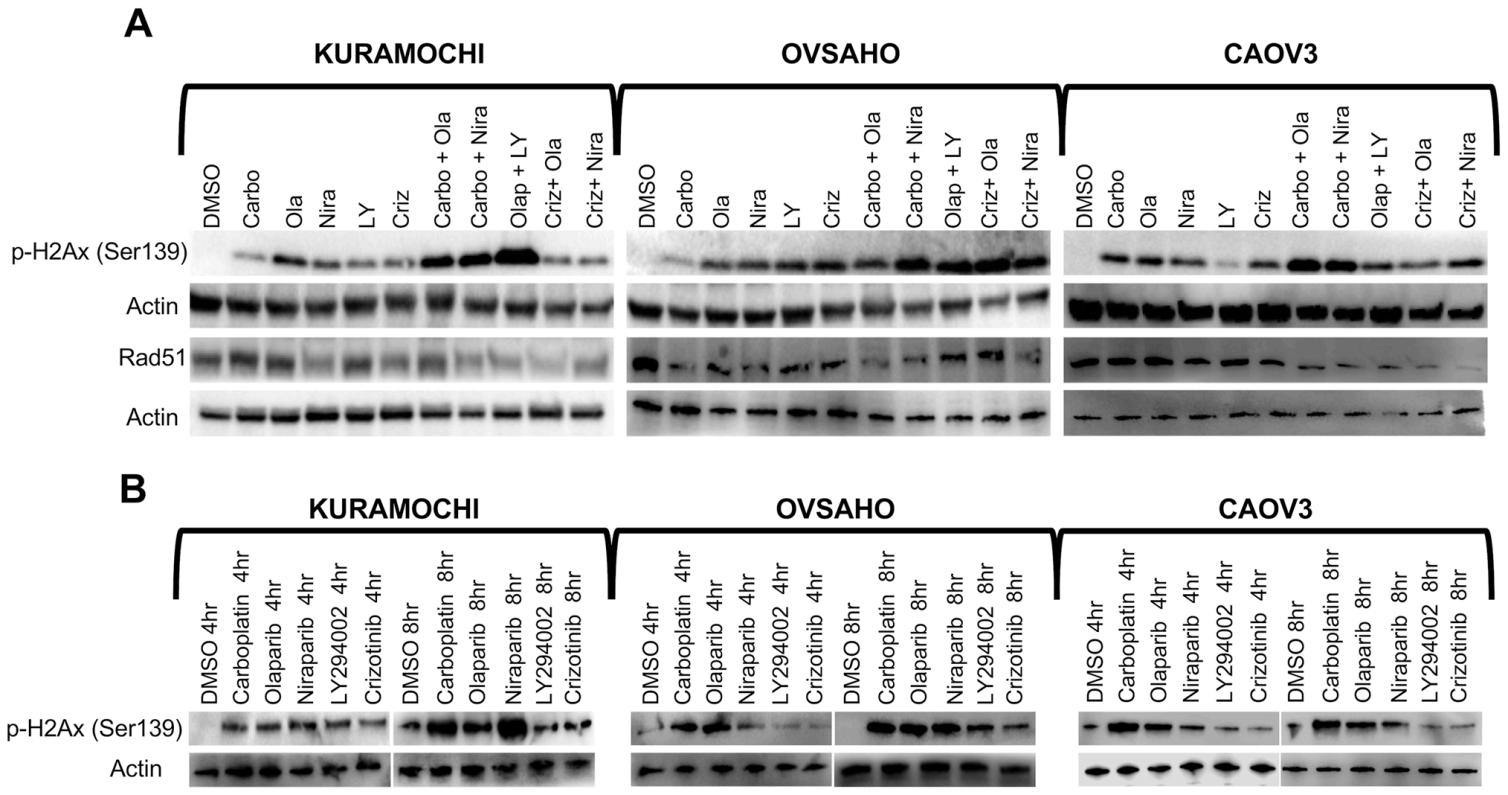
Accumulation of DNA double strand breaks. (**A**) HGSOC cells were treated with single compounds (IC_50_ concentrations) or sequential regimens for 1 week and analyzed by Western blot against *γ*H2AX and RAD51. (**B**) Western blot analysis of *γ*H2AX in cells treated with single agents for 4 h or 8 h (concentrations indicated in bold in Table 3).

Phosphorylation of histone H2AX (γH2AX) is a well-established marker of accumulation of dsDNA breaks [44]. We were specifically interested in assessing double-strand breaks (and not single-strand breaks which do not necessarily correlate with response to PARP inhibition) and therefore selected p-γ-H2AX as a specific marker of dsDNA breaks and RAD51 as a maker for DNA repair. HGSOC cells were treated for 4 h, 8 h or 1 week according to their IC_50_ concentrations (Table 2) and levels of γH2AX and RAD51 were investigated. Carboplatin induced an elevation of γH2AX levels at 4 h and 8 h, but the levels decreased again at 1 week. Moreover, the combination of a PARP inhibitor and crizotinib induced a prominent accumulation of dsDNA breaks, represented by elevation of γH2AX levels, in HGSOC cells at 72 h, which was also observed to a limited extent following LY294002 treatment (Figure 3A–3B, Supplementary Figure 2). The effect of PARP inhibitors with crizotinib was less dramatic at the shorter time points (4/8 h). Western blot analyses for induction of γH2AX accumulation upon treatment with monotherapies at concentrations selected from Table 3 (bold) were consistent with the immunofluorescence staining patterns (Figure 3 and Supplementary Figure 3). Carboplatin induced a slight decrease in RAD51 levels in HGSOC cells. On the other hand, crizotinib alone or in combination with either of the PARP inhibitors showed a significant decrease in the levels of RAD51 in all three HGSOC cell lines. Mono- and combination therapies including carboplatin and LY294002 with PARP inhibitors also showed effect of Rad51 phosphorylation (Figure 3A, Supplementary Figure 2).

### Cell cycle effects

**Figure 4:**
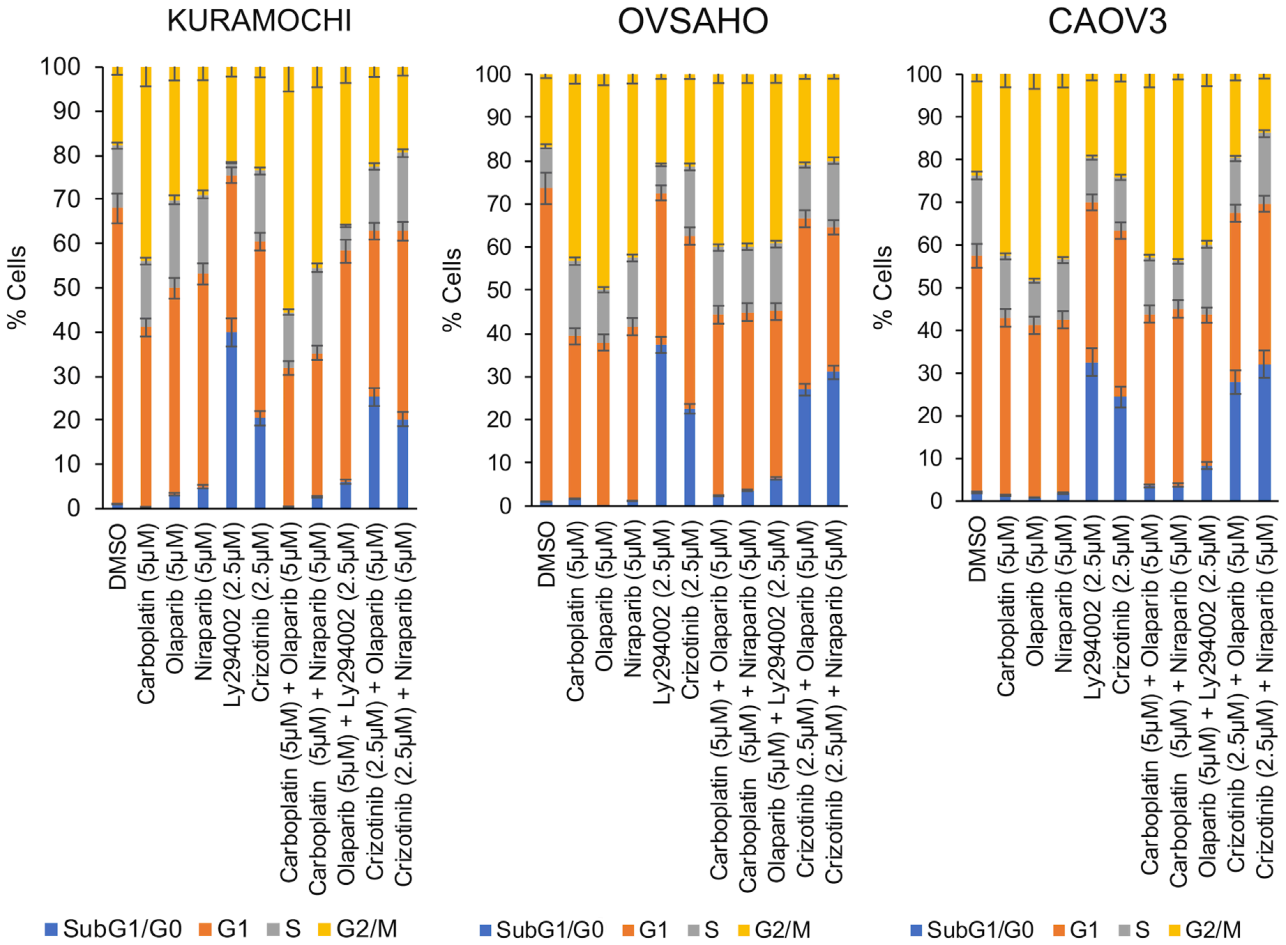
Induction of cell cycle arrest. Cells were either treated with single agents or with sequential combination treatments for 1 week (concentrations indicated in bold in Table 3).

Cell cycle analyses were performed by PI staining, followed by flow cytometry (drug concentrations used are indicated in bold in Table 3). Analyses revealed that monotherapies of carboplatin, olaparib and niraparib induced a G2/M cell cycle arrest whereas monotherapy with either LY294002 or crizotinib lead to accumulation of cells in sub-G1/G0 (Figure 4). The G2/M cell cycle arrest was maintained when carboplatin was combined with either of the PARP inhibitors. However, crizotinib alone and the combination of crizotinib with the PARP inhibitors predominantly favored the accumulation of cells in sub-G1/G0 (Figure 4), indicative of an increase in DNA fragmentation and suggesting apoptosis as the type of cell death induced. In contrast, sequential administration of olaparib followed by LY294002 predominantly caused a G2/M arrest and accumulation of a small cell population in sub-G1/G0 (Figure 4).

### Characterization of the cell death mechanism

**Figure 5:**
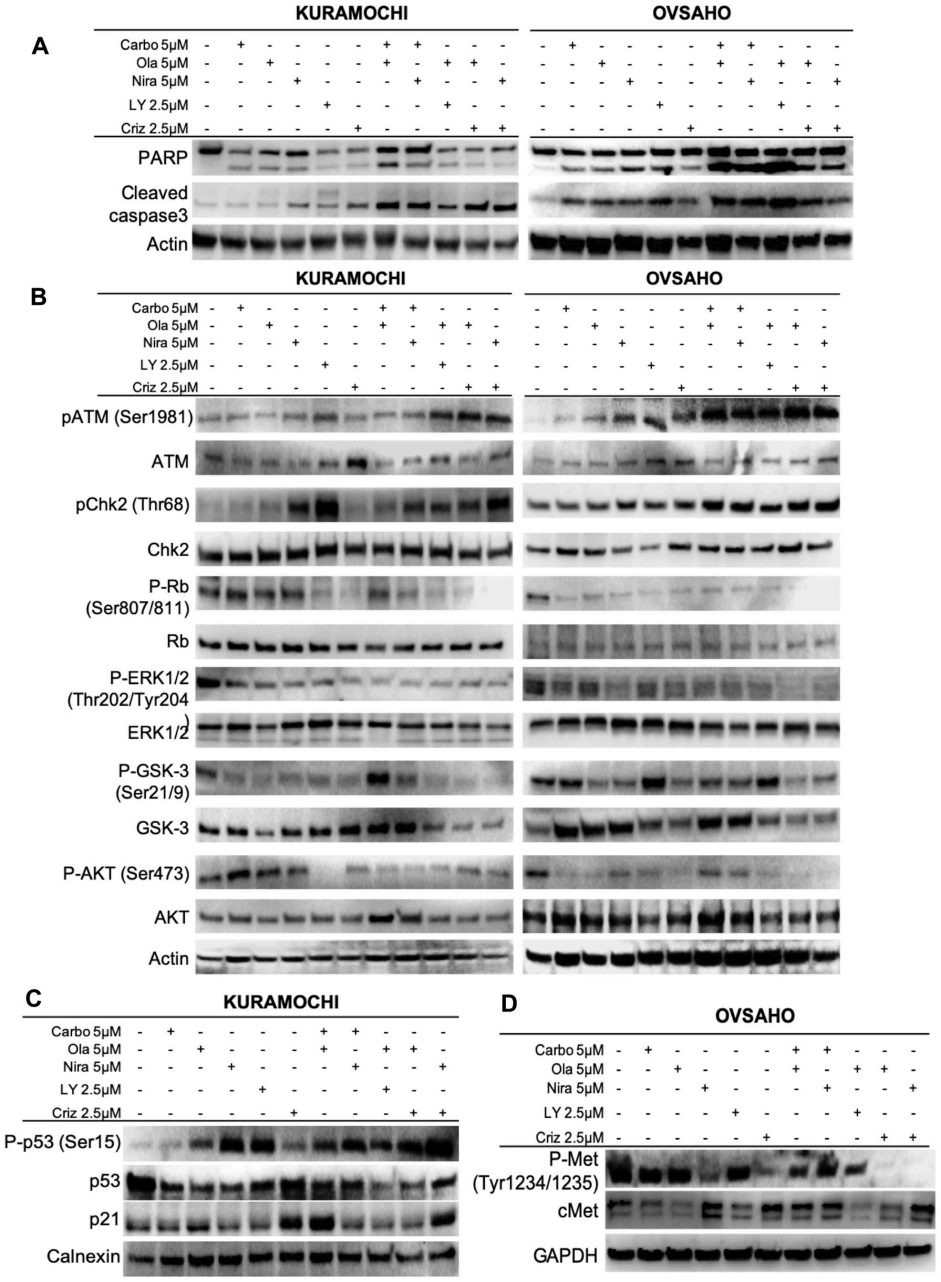
Effects on cellular and molecular pathways. Assessment of effects on apoptosis (**A**) and the ATM/CHK2 and c-Met pathways (**B**–**D**) after 1 week of single agent or sequential combination treatments.

We next investigated whether apoptosis was the cause of the cell death observed. The HGSOC cells were treated with either monotherapies or sequential regimens for 1 week (drug concentrations used are indicated in bold in Table 3). PARP, the downstream target of active cleaved caspase-3, is a well-established marker of apoptosis [45]. Western blot assays revealed that cleavage of both caspase-3 and PARP proteins increased significantly upon combination treatments and slightly after monotherapies (Figure 5A and Supplementary Figure 4), suggesting that the combinational approach potentiated caspase-dependent apoptotic cell death in HGSOC cells.

### The ATM-CHK2 pathway is activated upon PARP and c-Met inhibition

Western blot analyses were performed to further investigate the underlying mechanisms for the observed growth inhibition, cell cycle arrest and apoptosis induced by the drugs. Ataxia telangiectasia mutated (ATM) protein is a well-established kinase that is activated upon occurrence of DNA DSBs [46]. In addition, it has been shown that ATM protein activation and γH2AX foci formation, indicative of DSBs, increase upon PARP inhibition [47–49]. The results showed a significant increase in the phosphorylation of ATM (Ser1981), indicating its activation, in combination treatments of crizotinib and PARP inhibitors. Monotherapies did not cause any significant change in ATM phosphorylation (Figure 5B, Supplementary Figures 5–6). In parallel with ATM, sequential treatments induced phosphorylation of the downstream proteins CHK2 and p53 (Figure 5, Supplementary Figures 5–7). Based on these findings and the observed cell cycle arrest, we further explored the role of p21 and Rb. The results indicated that almost all the combination treatments as well as monotherapies induced dephosphorylation of Rb, thereby preventing its activation, but the effect was more severe in cells treated with the combination of PARP and c-Met inhibitors (Figure 5B, Supplementary Figures 5–6). In parallel, the levels of p21 protein were elevated (Figure 5C, Supplementary Figure 7B). We have focused on the (so far) novel finding of PARPi + crizotinib in the present study. Other combinations, while significant, have been previously shown and therefore we report statistical significance only for crizotinib combination treatments here.

### The c-Met pathway is targeted by co-administration of crizotinib and PARP inhibitors

**Figure 6:**
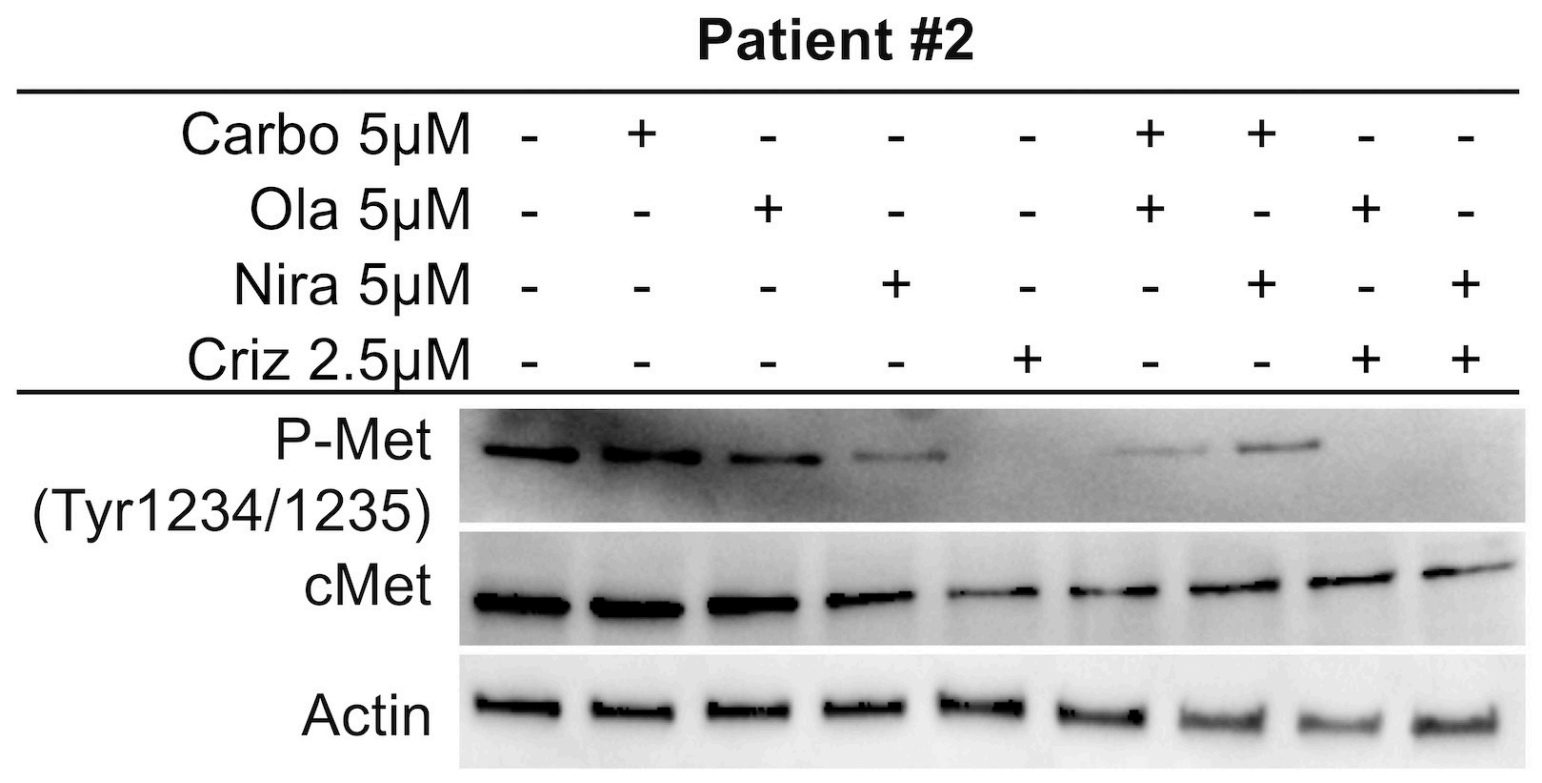
c-Met inhibition in Patient #2. Investigation of treatment effects on the c-Met pathway in the *ex vivo* patient sample after 1 week of single agent or sequential combination treatments.

Crizotinib inhibits anaplastic lymphoma kinase (ALK), c-ros oncogene1 (ROS1) and c-Met. From previous findings, it was suggested that crizotinib acts synergistically with cisplatin and induces apoptosis through the AKT and ERK pathways [50]. Another study proposed that c-Met may be a potential therapeutic target for ovarian cancer [51]. We first explored the inhibitory effect of crizotinib on c-Met in HGSOC cells. Results revealed that crizotinib, alone or in combination with a PARP inhibitor, attenuated the phosphorylation status of c-Met (Figure 5D and Supplementary Figure 7C). While co-treatments did not increase the effect on the c-Met pathway *per se* compared to crizotinib alone, the synergistic effect of co-targeting the c-Met pathway may potentiate the effect of the PARP inhibitors. Both monotherapies and combination treatments resulted in inhibition of the ERK1/2 and AKT proteins, reflected by a decrease in their phosphorylation status (Figure 5B and Supplementary Figures 5–6). GSK3 in contrast is inhibited by phosphorylation and displayed a decrease in its phosphorylated form upon monotherapy and combination treatments, indicating active GSK protein (Figure 5B and Supplementary Figures 4–5). We also investigated the effects on c-Met in patient #2. Results were in parallel with cell line experiments and co-administration of crizotinib and PARP inhibitors resulted in a decrease in phosphorylated active levels of c-Met (Figure 6, Supplementary Figure 7D). We performed these Western blot experiments with the CAOV3 cell line as well, but due to undetectable levels of endogenous p-cMet and potentially the presence of multiple mutations (including *TP53* and *ATM*) [43], these experiments were inconclusive.

## DISCUSSION

In recent years, PARP inhibitors have become of great interest for breast and ovarian cancer research and treatment. Several PARP inhibitors have been approved by the FDA and the European Medicines Agency for treatment of patients with platinum-sensitive, recurrent HGSOC with or without loss of *BRCA1/2* [20, 22, 23, 52–54] and studies reporting efficacy also in newly diagnosed patients, regardless of homologous-recombination deficiency status, are emerging [55, 56]. However, HGSOC is very aggressive and even though it generally responds to chemotherapy initially, patients tend to develop resistance rapidly [7]. While the introduction of PARP inhibitors has provided improvements in outcome, resistance is known to develop by various mechanisms, including reversion mutations [25–27].

In this study, we examined whether co-targeting of other signaling pathways important for tumor progression could potentiate the effect of PARP inhibition. We investigated sequential combinations of carboplatin, the PI3K/AKT/mTOR pathway inhibitor LY294002 and the c-Met inhibitor crizotinib with the PARP inhibitors olaparib or niraparib in HGSOC. Our data suggest that crizotinib was the most effective in potentiating the effect of the PARP inhibitors and provide insight into the cellular proteins/pathways affected by this combination treatment. The combination of LY294002 and niraparib was also tested, but it was found to be less potent in this setting and therefore not included in further analyses. Our results showed no PARP inhibitor or platinum response differences in cell lines depending on *BRCA2* status which confirms that HR deficiency can be due to mutations in genes other than *BRCA1/2*. In addition, niraparib has been approved also for non-*BRCA*-mutated cancers, in line with a treatment benefit also among these patients [21, 57, 58].

**Figure 7:**
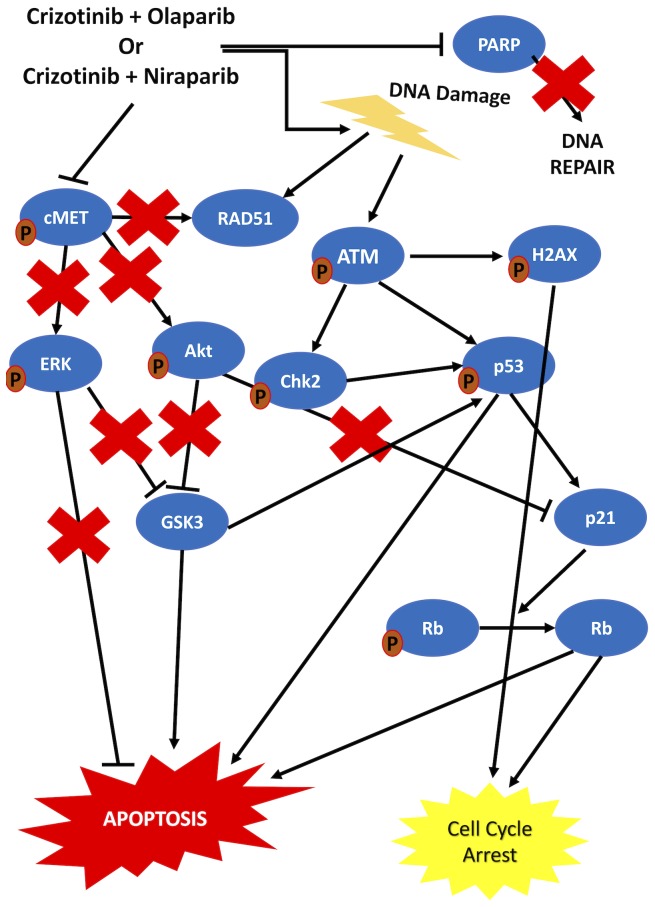
Schematic representation of the proposed mechanism of action of the combination treatments. Sequential combination treatment with crizotinib and PARP inhibitors was shown to be more effective than the combination of Carboplatin and PARP inhibitors. Deregulation of multiple proteins including ERK, Akt and p53, all contributing to cell cycle arrest and apoptosis, was induced by the sequential combination of crizotinib and PARP inhibitors in HGSOC cells.

Previous data suggest that dsDNA break formation is stimulated and the ATM pathway is activated upon PARP inhibition [15, 46–48, 59]. Our results are in line with these data, indicating that γH2AX foci formation increased and ATM phosphorylation levels were elevated upon combination treatment. On the other hand, it was previously proposed that c-Met inhibition downregulated RAD51 and hence sensitized tumor cells to DNA damaging agents [60]. Our data also suggested a slight decrease in RAD51 in cells treated with carboplatin and importantly a significant decrease in cells treated with crizotinib or the combinations. This finding in combination with the literature demonstrates the role of cMet inhibiton in sensitizing HGSOC cells to PARP inhibition. It has further been proposed that DNA DSBs induce ATM phosphorylation, which leads to phosphorylation of p53 and CHK2. p53 activation induces p21 which activates Rb, thereby suppressing cell cycle activity and proliferation [61–65]. Our results indicate an increase in the phosphorylation of both p53 and CHK2 upon co-targeting of c-Met and PARP. Furthermore, p21 levels increased concomitantly with a decrease in the phosphorylated form of Rb in co-treated cells compared to control cells and activation of Rb induced cell cycle arrest. In addition, we confirmed the inhibition of c-Met by crizotinib, as indicated by a decrease in its phosphorylated form. This inhibition was maintained upon combination treatment with the PARP inhibitors. The literature suggests that when c-Met is active, it induces Akt and ERK protein activity [66, 67]. GSK3 is negatively regulated by Akt and ERK proteins via phosphorylation at Ser21/9, thereby inducing cell survival and inhibition of apoptosis [68–70]. GSK3B has been suggested to bind and activate p53 [71]. Our results indicate that upon combination treatment, Akt and ERK proteins were inhibited, resulting in the activation of GSK3, which contributed to the activation of p53 and subsequent induction of apoptosis. The observed increase in cleaved caspase-3 and PARP implies apoptosis as the predominant mechanism of cell death. Taken together, our data suggest that sequential treatment with crizotinib and PARP inhibitors induces cell cycle arrest and apoptosis via targeting of the c-Met/AKT/MAPK and ATM/CHK2 pathways in HGSOC cells (Figure 7). Importantly, treatment effects in primary human HGSOC cells from ascites fluid mirrored the findings established in HGSOC cell lines.

Resistance towards PARP inhibitors is frequently observed in c-Met overexpressing cells. This resistance is thought to arise due to phosphorylation of PARP via c-Met, thereby preventing binding of PARP inhibitors [72]. Our results indicating a synergistic effect of co-targeting c-Met and PARP may suggest a novel approach to overcoming resistance towards PARP inhibitors in HGSOC, which warrants further investigation. Taken together, c-Met inhibitors such as crizotinib may be considered together with PARP inhibitors for further development as a novel treatment strategy for patients diagnosed with recurrent HGSOC. Of note, crizotonib and PARP inhibitors are used differently in the clinic. Whereas crizotonib is approved as an upfront treatment (1st line monotherapy in non-small cell lung cancer), the PARP inhibitors are thus far approved as maintenance treatments. The results from two randomized trials evaluating PARP inhibitors as active treatment options instead of chemotherapy have recently been made public. The NSGO-AVANOVA/ENGOT-ov24 trial showed a better outcome for patients treated with niraparib plus bevacizumab *vs.* niraparib alone, regardless of *BRCA1/2* mutation status [73], and the SOLO3 trial showed a better outcome for patients treated with olaparib monotherapy *vs.* non-platinum containing chemotherapy in germline *BRCA1/2*-mutated patients, both in platinum-sensitive, recurrent ovarian cancer (Clinicaltrials.gov identifier NCT2282020) [74]. If PARP inhibitors are approved as an active treatment option rather than as maintenance treatment following chemotherapy in the future, the effect may be enhanced when combined with crizotinib. Pre-clinical combination experiments as described herein are highly relevant for exploring novel treatment strategies and elucidating the mechanisms involved. Nevertheless, functional *in vivo* studies and clinical trials will be required before these new regimens can be introduced in the clinic.

## MATERIALS AND METHODS

### Drugs

Carboplatin (S1215), olaparib (AZD2881) (S1060), niraparib (MK4827) (S7625), LY294002 (S1105), and crizotinib (S1068), were purchased from Selleck Chemicals (Houston, TX).

### Cell culture

HGSOC cell lines OVSAHO (*BRCA2* homozygous deletion), KURAMOCHI (*BRCA2* mutant) and CAOV3 (*BRCA1/2* wildtype) were cultured in RPMI 1640 medium or DMEM/High Glucose medium with 10% Fetal Bovine Serum (FBS), 100 units/mL penicillin/streptomycin (Hyclone, Logan, UT, USA). The human ovarian clear cell carcinoma (OCCC) cell line (JHOC5) was cultured in DMEM:HAMF12 1:1 with 10% FBS, 1% non-essential amino acid (NEAA), and 100 units/mL penicillin/streptomycin (Hyclone). MCF10A cells were cultured in DMEM/F-12 medium (1:1) supplemented with 5% FBS, 100 units/mL penicillin/streptomycin (Hyclone), 100 ng/mL cholera toxin, 10 ng/mL epidermal growth factor, 0.5 μg/mL hydrocortisone, and 10 μg/mL insulin (Sigma Aldrich). KURAMOCHI and OVSAHO cell lines were purchased from the Japanese Collection of Research Bioresources (JCRB) Cell Bank (Osaka, Japan). JHOC5 was obtained from the RIKEN National Bio-Resource Center (Ibaraki, Japan), and CAOV3 and MCF10A cells were purchased from ATCC (Manassas, VA, USA). Mycoplasma testing was performed for all the cell lines used.

Primary cells obtained from ascites fluid of patients (*n* = 2) diagnosed with HGSOC were cultured in DMEM/High Glucose medium with 10% FBS and 1x anti-anti. The ascites from patient #1 (stage IVA) was obtained during primary upfront surgery and contained malignant (metastatic) cells. Thus, the patient was chemo-naïve at the time of drainage. Post-operatively the patient received six-cycles of platinum-based combination chemotherapy and 10 cycles of bevacizumab before progression. Patient #2 was diagnosed with primary inoperable HGSOC with wide-spread abdominal metastases (stage X). She was scheduled for six treatment cycles, but received five cycles of carboplatin upfront; the treatment was however discontinued prematurely due to side effects. Disease progression was later observed. The patient was considered platinum-sensitive, but due to remaining, disabling side effects received 2nd line treatment with weekly paclitaxel. Ascites was drained when she had recently started 2nd line treatment. The patient had widespread, abdominal tumor dissemination. Thus, the ascites was considered malignant (metastatic).

### NCI-sulforhodamine B assay for *in vitro* cytotoxicity screening

Cell lines or primary cells were treated with increasing concentrations of the single compounds (2.5–40 μM) for 1 week. The cells were fixed with 10% (v/v) Trichloroacetic acid (Sigma Aldrich) and stained with 0.4% (m/v) of Sulforhodamine B (Sigma Aldrich) in 1% acetic acid. The absorbance values were obtained at 515 nm. All experiments were performed in triplicate and repeated at least three independent times.

For combination treatments, a sequential regimen (treatment with one compound for 72 h followed by a combination of two inhibitors for a further 96 h) was explored. Negative controls fallowed the same combination regimen (72 h + 96 h). The Combination Index (CI) values were obtained using the Chou-Talalay method [75]. CI values = 1, >1, and <1 represents additive, antagonistic and synergistic interactions respectively [76, 77].

### Real-time cell electronic sensing (xCeLLigence)

Cells were inoculated into 96x E-plates (5,000 cells/well) and were monitored every 30 minutes using the real-time cell electronic sensoring system (RT-CES, ACEA, Champaign, IL, USA). 24 hours after seeding, cells were treated with the compounds (2.5–80 μM). Each experiment was repeated at least three times. The electronic readout (cell-sensor impedance) was displayed as an arbitrary unit, the cell index. The cell index value was noted every 10 minutes for the first 24 h and then every 30 minutes. The cell inhibition rate (%) = [1–(CellIndex_treated cells_/CellIndex_DMSO_)]×100.

### γH2AX (Ser139) immunofluorescence staining

HGSOC cell lines were inoculated in 6-well plates for 24 h, followed by treatment with IC_50_ concentrations of the compounds or DMSO control for 72 h. Cells were fixed with cold methanol for 30 minutes, permeabilized with 2% NP40 for 20 minutes and blocked with 2% BSA in 1xPBS (0.1% Tween). The anti-γH2AX (Ser139) (Cell Signaling, Denvers, MA, USA) antibody was prepared 1:200 in 2% BSA in 1xPBS (0.1% Tween) and applied for 2 hours. The secondary antibody Alexa488 (rabbit IgG) (Cell Signaling) was prepared 1:1000 in 2% BSA in 1xPBS (0.1% Tween) and applied for 1 hour. DAPI mounting media was used to stain the nuclei and to mount the samples. The samples were observed under an Olympus fluorescence microscope. Quantification of the images was performed with ImageJ software; 50 nuclei were quantified per cell line/treatment.

### Cell cycle analysis

Cells were inoculated for 24 hours and treated with either single compounds or with sequential combinations (as outlined above). Cells were fixed in ice-cold 70% ethanol and cell pellets were resuspended in propidium iodide (PI) solution (50 µg/ml PI (Sigma–Aldrich), 0.1 mg/mL RNase A, 0.05% Triton X-100, and ice-cold 1xPBS) for 40 min at 37°C in the dark. After centrifugation, the pellets were re-suspended in PBS and cell cycle analysis was conducted with the FACS Verse (BD Biosciences Immunocytometry Systems, San Jose, CA, USA).

### Western blot analysis

HGSOC cell lines were cultured for 24 h and treated with monotherapies or sequential regimens as outlined above (for 1 week, 4 hours or 8 hours). Following incubation of the membranes in blocking solution (5% milk powder in 1 TBS-T (0.1% Tween)), membranes were incubated with primary antibodies: anti-γH2AX (Ser139) (Cell Signaling, 2577), RAD51 (Abcam, ab213), anti-PARP (Cell Signaling, 9532), anti-p-Akt (Ser473) (Cell Signaling, 9271), anti-Akt (Cell Signaling, 9272), anti-cleaved-caspase3 (Cell Signaling, 9661), anti-c-Met (Cell Signaling, 8198), anti-p-Met (Tyr1234/1235) (Cell Signaling, 3077), anti-ATM (Cell Signaling, 92356), anti-p-ATM (Ser1981) (Cell Signaling, 5883), anti-p53 (Cell Signaling, 9282), anti-p-p53 (Ser15) (Cell Signaling, 9286), anti-Chk2 (Cell Signaling, 2662), anti-p-Chk2 (Thr68) (Cell Signaling, 2661), anti-Rb (Cell Signaling, 9309), anti-p-Rb (Ser807/811) (Cell Signaling, 8516), anti-Erk (Santa Cruz, sc292838), anti-p-Erk (Thr202/Tyr204) (Cell Signaling, 4370), anti-GSK3 (Cell Signaling, 9338), anti-p-GSK3 (Ser21/9) (Cell Signaling, 8566), p21 (Cell Signaling, 2947), on a shaker over night at 4°C. Secondary antibodies anti-rabbit (Sigma, A6154) and anti-mouse (Sigma, A0168) were applied in 1:5000 ratio in 5% BSA-TBS-T (0.1%) for one hour at room temperature. Actin (Cell Signaling, 4967) and GAPDH (Cell Signaling, 5174) primary antibodies [1:5000 dilutions in 5% milk-powder in TBS-T (0.1% Tween)] were used to ensure equal protein loading on gels. For visualization of the results, chemoluminescence was performed with the ECL kit according to the manufacturer’s protocol (Bio-Rad, Hercules, CA, USA). Quantification of the images was performed with ImageJ software. Phosphorylation assessment was calculated as ((p-protein/control)/(total protein/control) and non-phosphorylated proteins were calculated as protein/control.

### Statistical analyses

Statistical comparisons for differences in the mean effects between treatments were conducted with two-tailed student’s *t*-test with unequal variances using Microsoft Excel. (^*^ *p* < 0.05; ^**^ *p* < 0.01; ^***^ *p* < 0.005). IC_50_ values calculated are presented as mean ± error as well as the graphs in the figures. Experiments were repeated three times (*n* = 3). For statistical analysis of quantification of the Western blot images, ANOVA was performed followed by Tukey’s test to assess significance. In all figures the error bars represent standard error of the mean (SEM).

## SUPPLEMENTARY MATERIALS



## Abbreviations

EOC: Epithelial ovarian cancer
HGSOC: high-grade serous ovarian cancer
ssDNA: single-strand DNA
PARP: Poly(ADP-ribose) polymerase
BER: base excision repair
dsDNA: double-strand DNA
HR: homologous recombination
NHEJ: non-homologous end joining
FDA: US Food and Drug Administration
MET: mesenchymal-epithelial transition factor
SRB: Sulforhodamine B
CI: Combination Index
RT-CES: real-time cell electronic sensoring system
DMSO: dimethyl sulfoxide
FACS: fluorescence-activated cell sorting
PI: propidium iodide
ATM: Ataxia telangiectasia mutated
Rb: retinoblastoma
ALK: anaplastic lymphoma kinase
ROS1: c-ros oncogene1
