# Supplementary material for: Crizotinib and PARP inhibitors act synergistically by triggering apoptosis in high-grade serous ovarian cancer
Source: Oncotarget. 2019 Dec 10;10(65):6981–96. doi: 10.18632/oncotarget.27363 (PMC6916751; doi:10.18632/oncotarget.27363)
Supplement: Supplementary file 1 [file oncotarget-10-6981-s001.pdf]

Crizotinib and PARP inhibitors act synergistically by triggering apoptosis in high-grade serous ovarian cancer

SUPPLEMENTARY MATERIALS

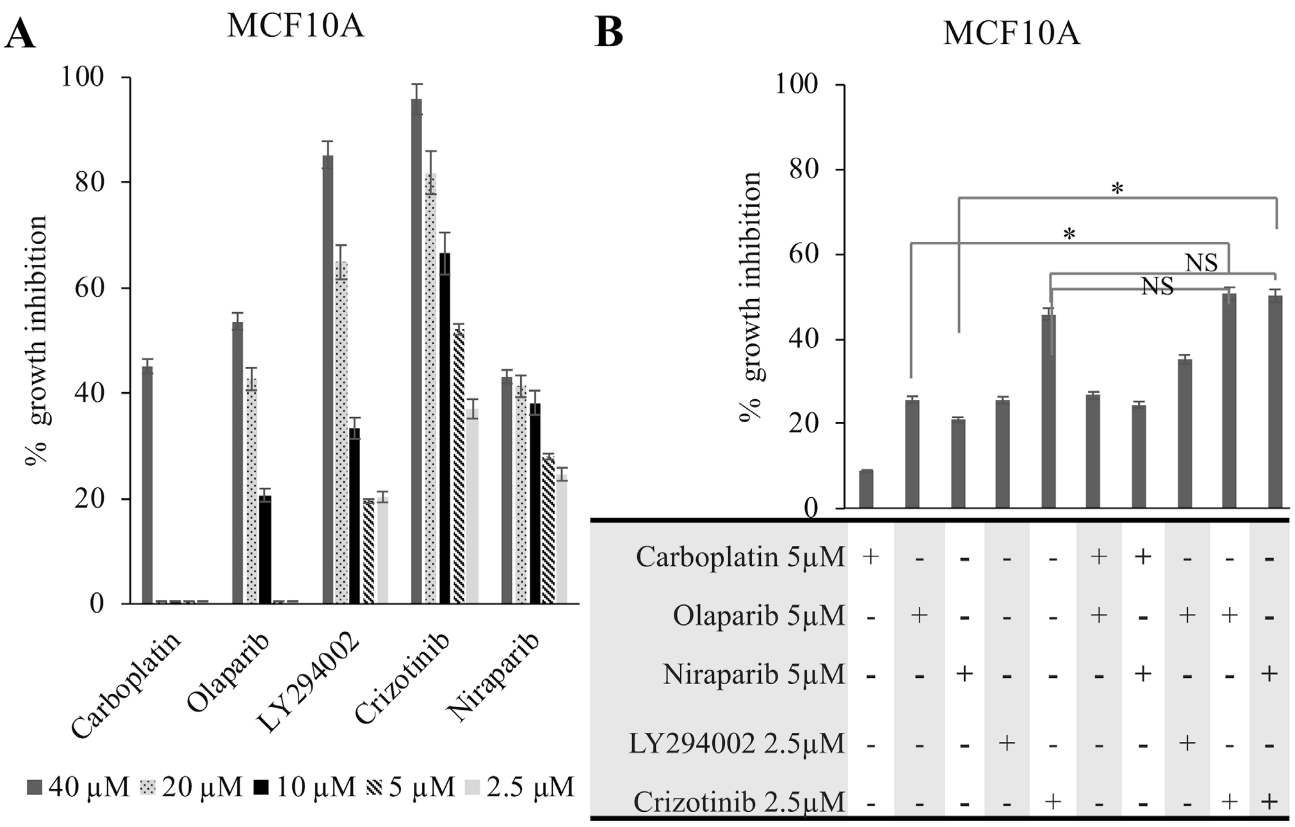

Supplementary Figure 1: Growth inhibitory effects of (**A**) single agent and (**B**) combination regimens on non-tumorigenic breast epithelial cells. MCF10A cells were treated with single agents or sequential regimens for 1 week and growth inhibitory effects were measured by the NCI-SRB assay. All experiments were conducted in triplicates. Statistical analysis of mean values ($n = 3$) was performed with student's t-test ($^{*}p < 0.05$; $^{**}p < 0.01$; $^{***}p < 0.005$; NS, not significant).

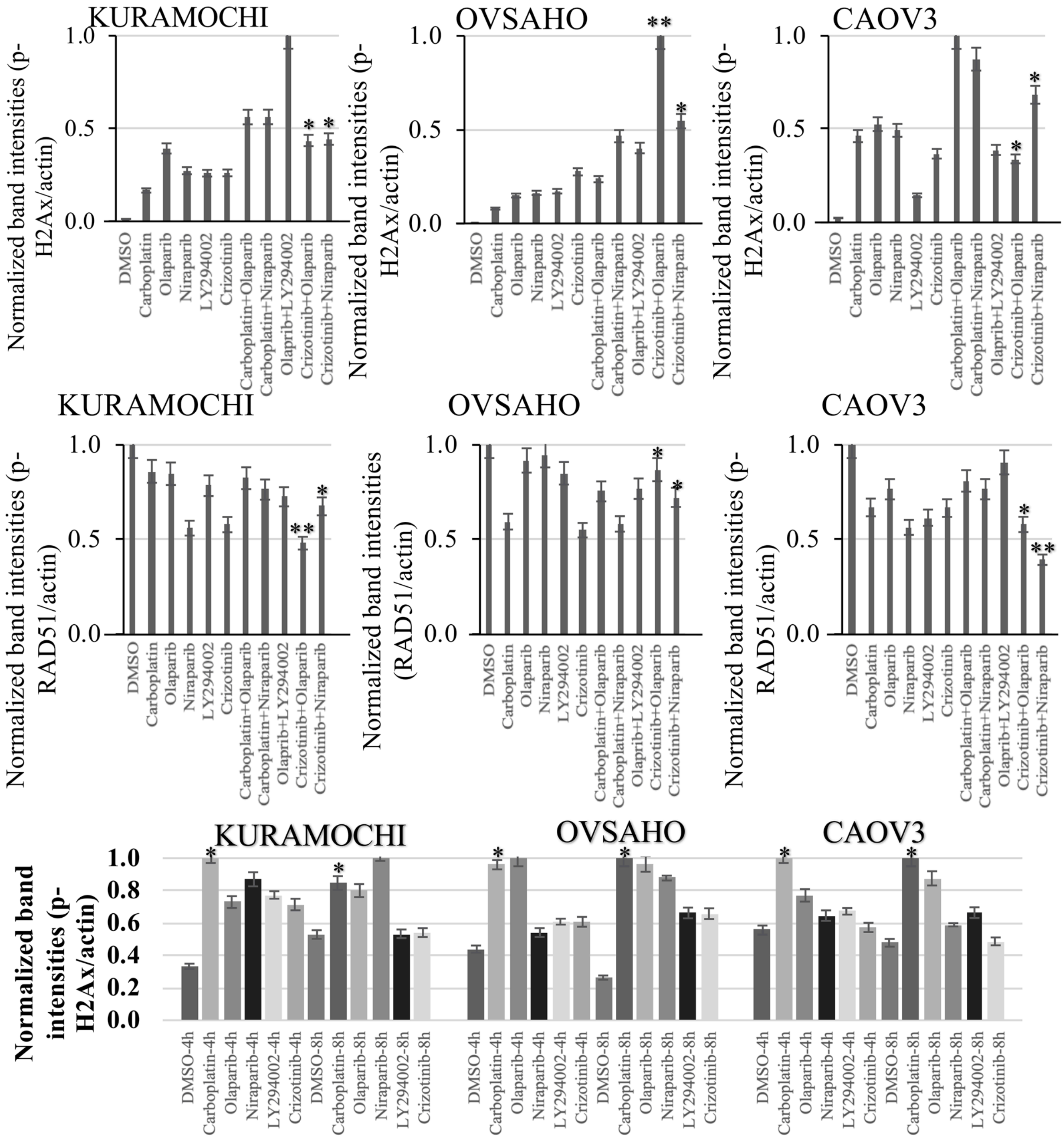

Supplementary Figure 2: Quantification of Western blot analysis of γH2AX and RAD51. Cells treated with single agents or sequential regimens for 1 week (concentrations indicated in bold in Table 3). Quantification of the images was performed with ImageJ software. ($^{*}p < 0.05$; $^{**}p < 0.01$; $^{***}p < 0.005$).

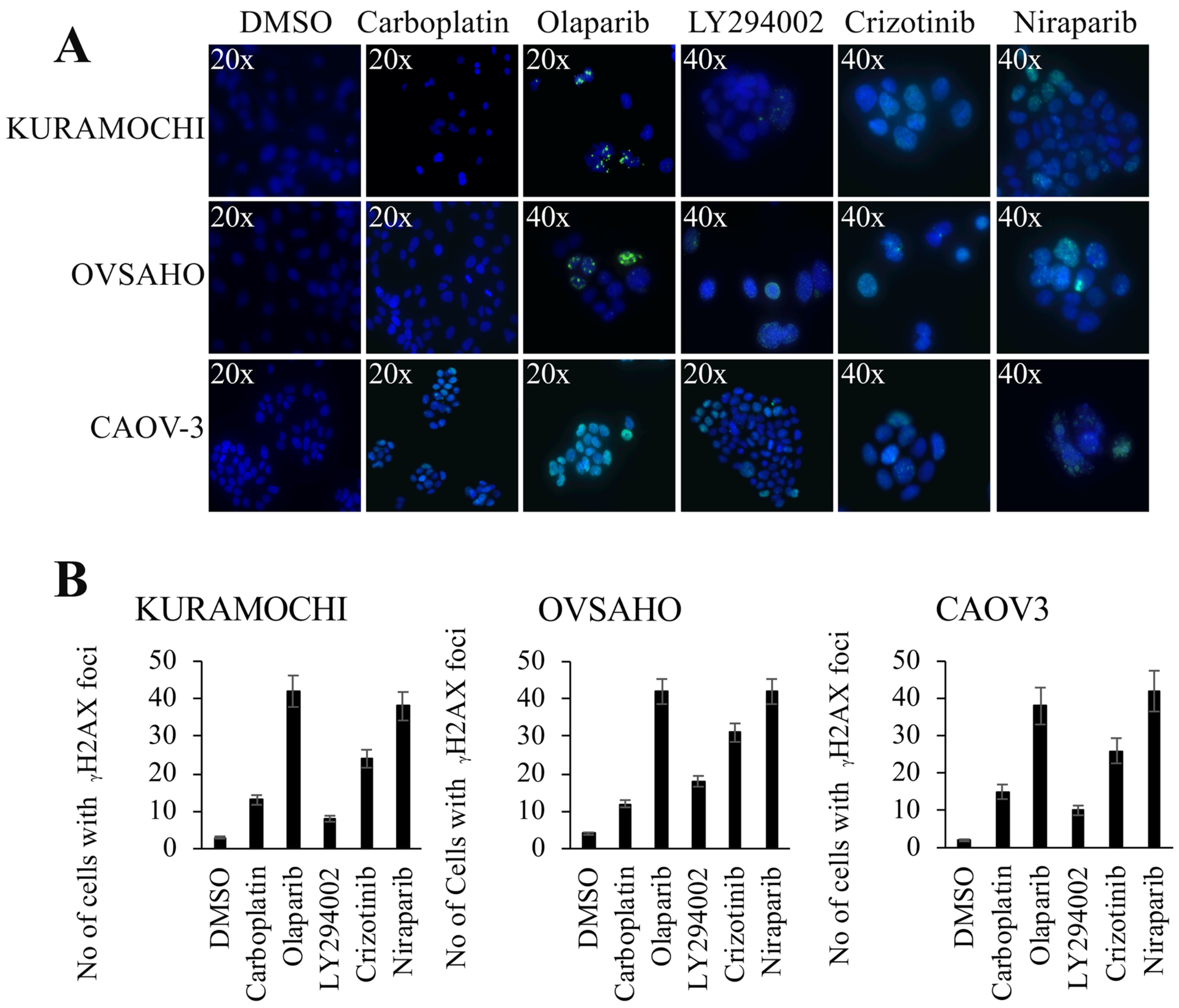

Supplementary Figure 3: Immunoflorescence staining of γH2AX. (**A**) Cells treated with single agents for 72 h (concentrations indicated in bold in Table 3) were analyzed by immunoflorescence staining of γH2AX (green). DAPI (blue) was used to visualize nuclei. DMSO was used as a negative control. (**B**) The bar graphs represent the quantification of γH2AX fluorescence intensity averaged from >50 nuclei processed by ImageJ.

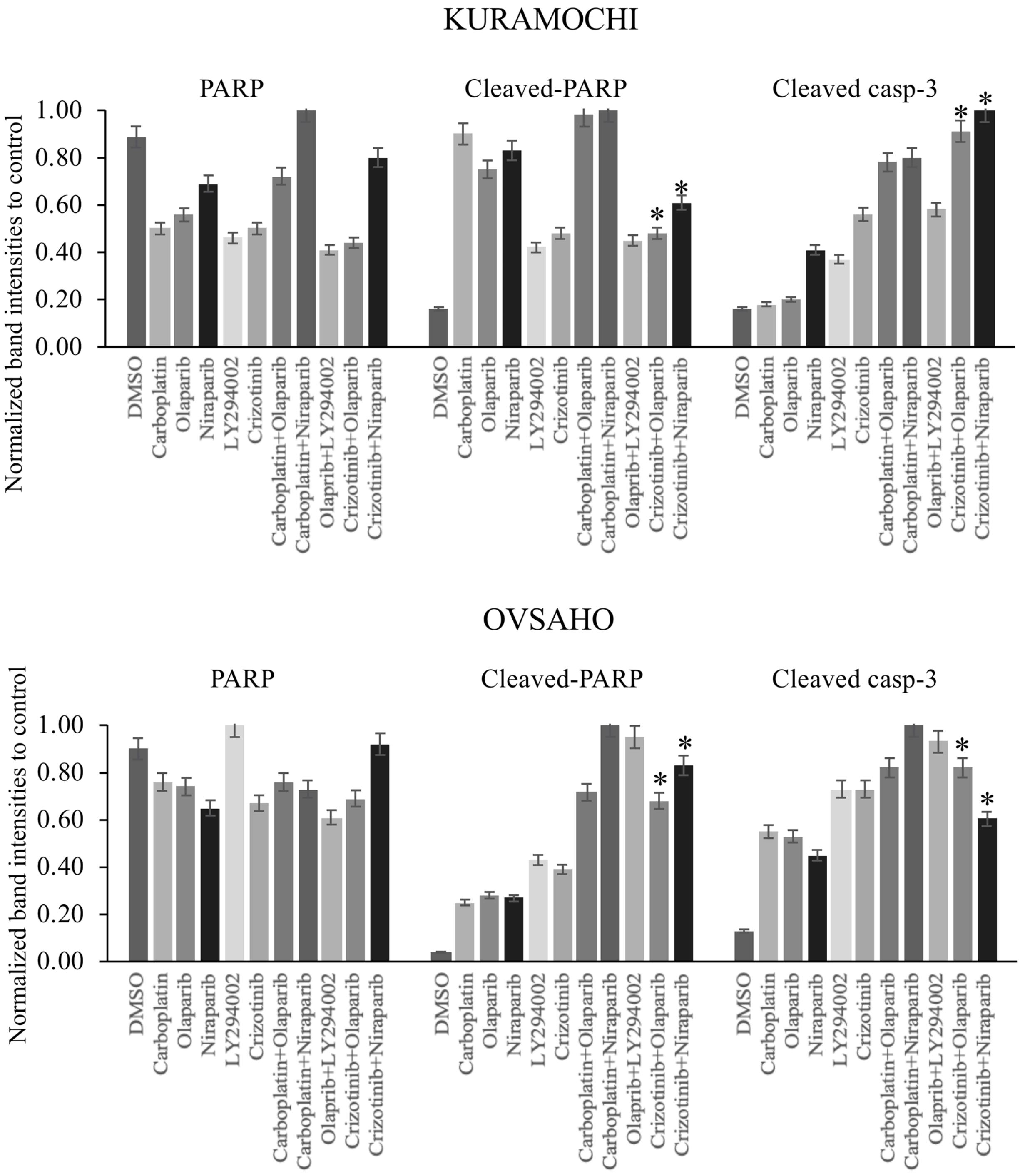

Supplementary Figure 4: Effects on cell death pathways. Assessment of effects on PARP, cleaved PARP and cleaved caspase 3 proteins after 1 week of single agent or sequential combination treatments of KURAMOCHI and OVSAHO cell lines. Quantification of the Western blot images with ImageJ ($^{*}p < 0.05$; $^{**}p < 0.01$; $^{***}p < 0.005$ significance values calculated compared to DMSO controls using ANOVA followed by Tukey's test).

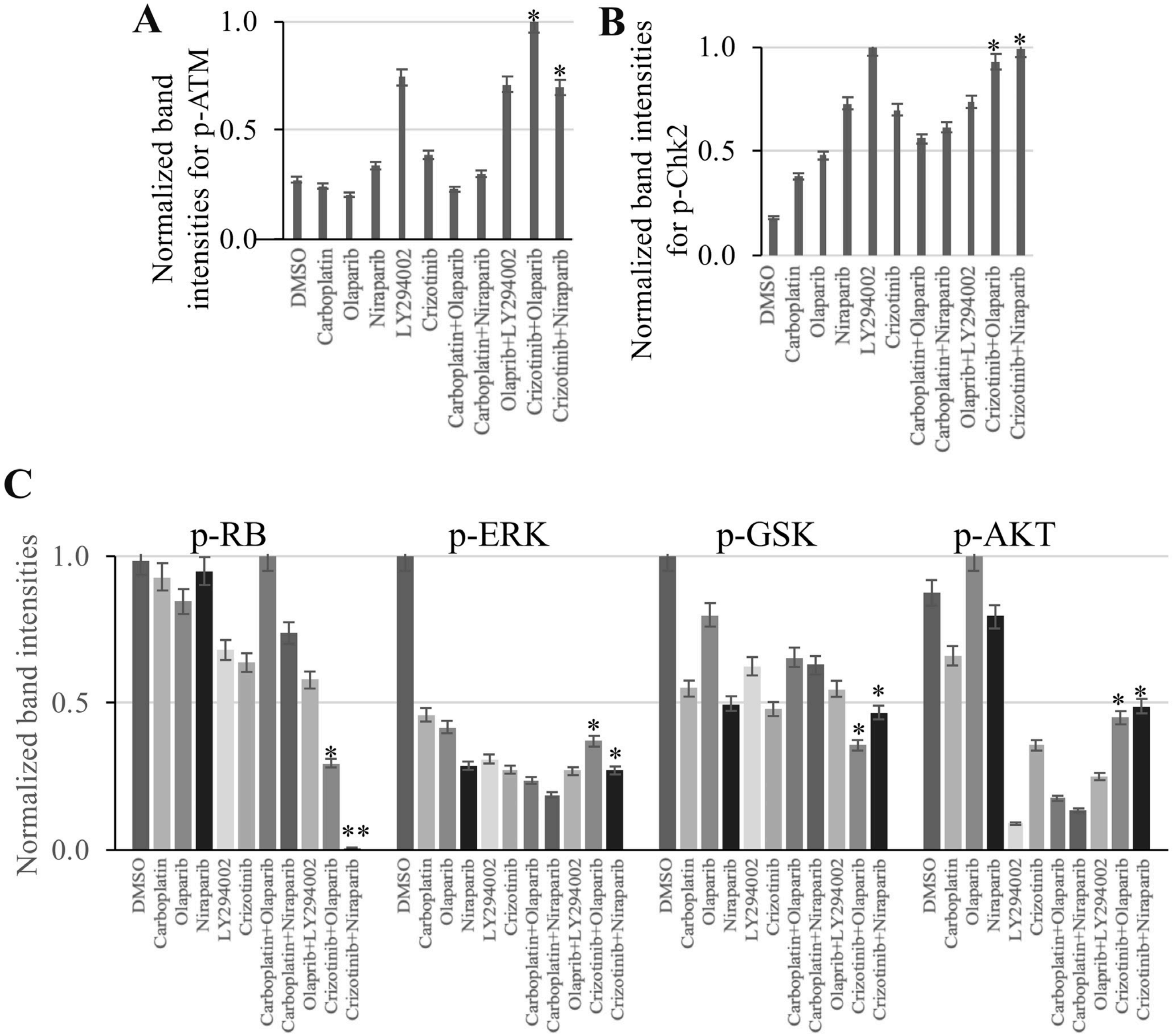

Supplementary Figure 5: Effects on cellular and molecular pathways. Investigation of effects of the drugs on (**A**) ATM, (**B**) Chk2, (**C**) (Rb, Erk, GSK and AKT) pathways after 1 week treatment of KURAMOCHI cell line. Quantification of Western blot images performed with ImageJ software (p-protein/control)/(total-protein/control). ($^{*}p < 0.05$; $^{**}p < 0.01$; $^{***}p < 0.005$ significance values were compared to DMSO control).

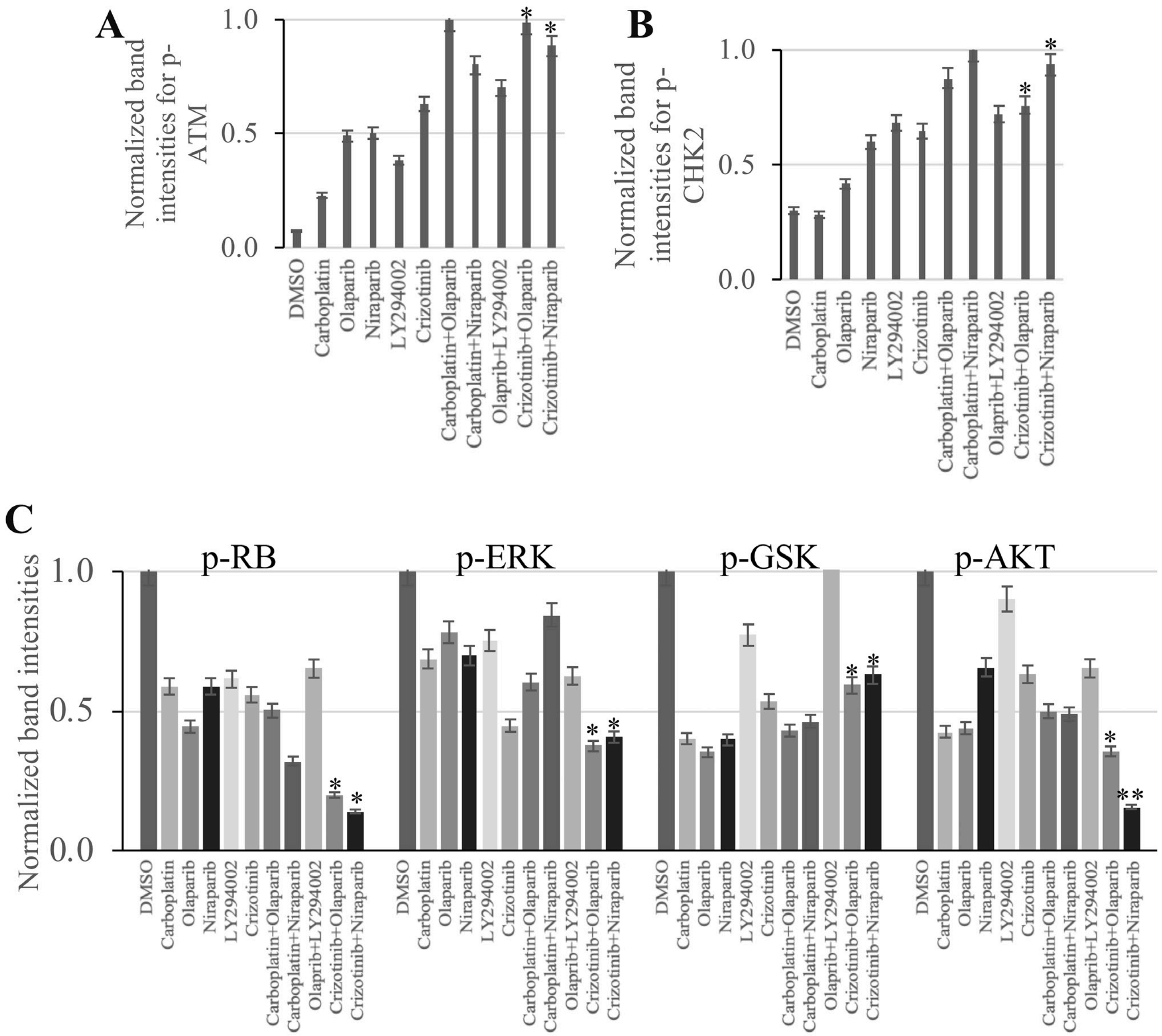

Supplementary Figure 6: Effects on cellular and molecular pathways. Investigation of effects of the drugs on (**A**) ATM, (**B**) Chk2, (**C**) (Rb, Erk, GSK and AKT) pathways after 1 week treatment of OVSAHO cell line. Quantification of Western blot images performed with ImageJ software (p-protein/control)/(total-protein/control). ($^{*}p < 0.05$; $^{**}p < 0.01$; $^{***}p < 0.005$ significance values were compared to DMSO control).

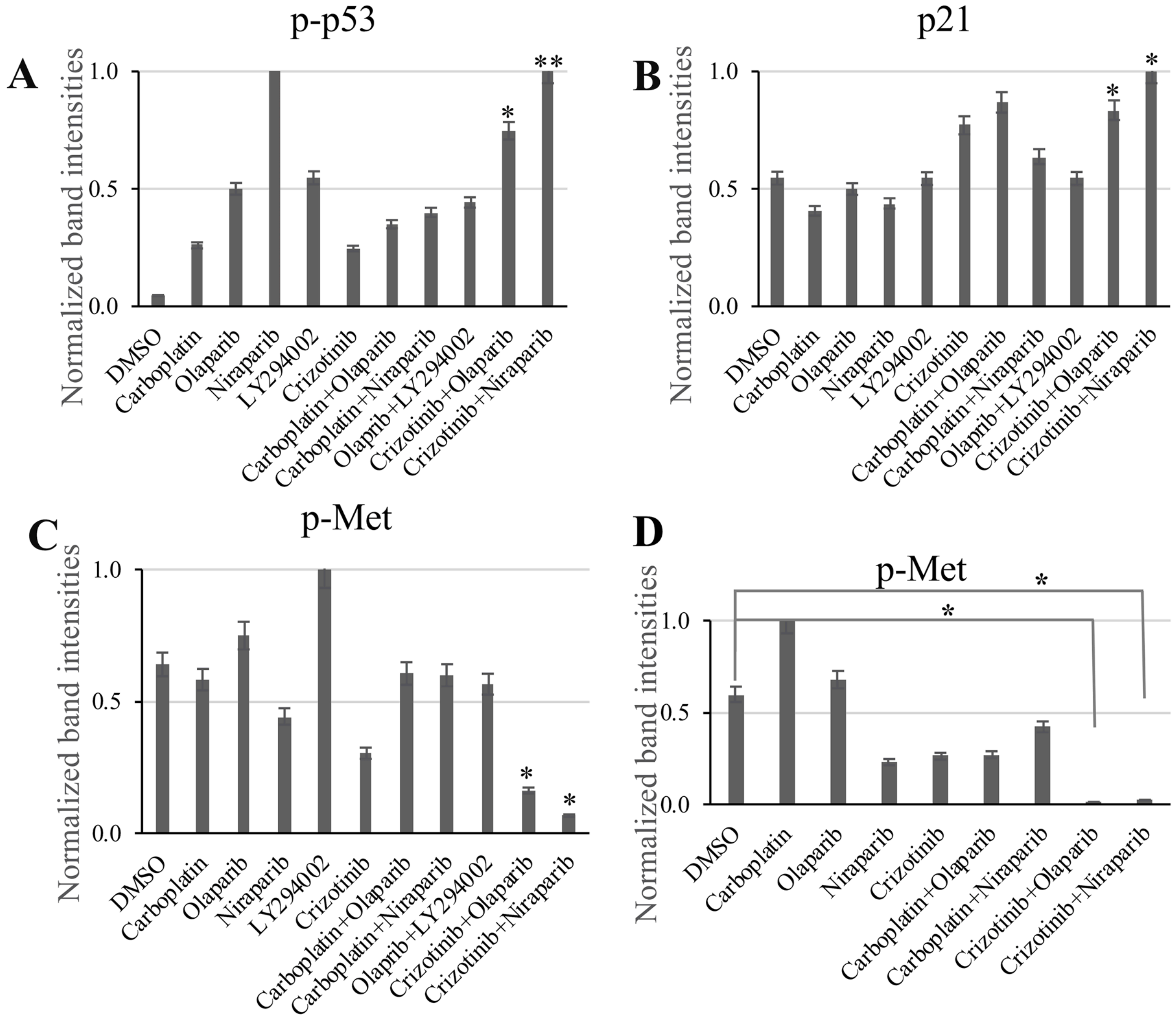

Supplementary Figure 7: Effects on cellular and molecular pathways. Investigation of effects of the drugs on (**A**) p53, (**B**) p21 and (**C**) c-Met pathways on cancer cell lines and (**D**) an *ex vivo* patient sample after 1 week of treatment. Quantification of Western blot images performed with ImageJ software (p-protein/control)/(total-protein/control) (or for p21: (protein/control)). ($^{*}p < 0.05$; $^{**}p < 0.01$; $^{***}p < 0.005$ significance values were compared to DMSO control).
